# Supplementary material for: Pervasive allele-specific regulation on RNA decay in hybrid mice
Source: Life Sci Alliance. 2018 May 16;1(2):e201800052. doi: 10.26508/lsa.201800052 (PMC6238540; doi:10.26508/lsa.201800052)
Supplement: Supplementary file 1 [file LSA-2018-00052_TableS1.docx]

**Supplemental Table 1. Statistics of allelic read mapping**

| Samples | Total read pairs [million] | Read pairs after trimming [million] | Read pairs concordantly mapped to autosome [million (%)] **^1)^** | Read pairs assigned to C57BL/6J [million (%)] **^2)^** | Read pairs assigned to SPRET/EiJ [million (%)] **^2)^** | Read pairs which can not be assigned [million (%)] **^2)^** |
| --- | --- | --- | --- | --- | --- | --- |
| ActD 0h rep1 | 127.8 | 123.8 | 93.0 (75.1%) | 31.4 (33.7%) | 29.2 (31.4%) | 32.4 (34.9%) |
| ActD 0h rep2 | 130.9 | 126.8 | 96.9 (76.5%) | 32.4 (33.5%) | 30.3 (31.3%) | 34.2 (35.2%) |
| ActD 0.5h rep1 | 120.9 | 117.1 | 89.5 (76.4%) | 29.7 (33.1%) | 27.7 (30.9%) | 32.2 (36.0%) |
| ActD 0.5h rep2 | 129.4 | 125.3 | 97.6 (77.9%) | 32.7 (33.6%) | 30.6 (31.3%) | 34.3 (35.1%) |
| ActD 1.5h rep1 | 134.3 | 130.0 | 100.6 (77.4) | 33.1 (33.0%) | 30.9 (30.7%) | 36.5 (36.3%) |
| ActD 1.5h rep2 | 137.1 | 132.3 | 103.6 (78.0%) | 34.5 (33.3%) | 32.2 (31.1%) | 36.8 (35.6%) |

1. Percentages are calculated as fractions over the number of read pairs after trimming.
2. Percentages are calculated as fractions over the number of read pairs concordantly mapped to autosome.
