# Supplementary material for: Pervasive allele-specific regulation on RNA decay in hybrid mice
Source: Life Sci Alliance. 2018 May 16;1(2):e201800052. doi: 10.26508/lsa.201800052 (PMC6238540; doi:10.26508/lsa.201800052)
Supplement: Supplementary file 2 [file LSA-2018-00052_TableS2.docx]

**Supplemental Table 2. PCR primers for PacBio validation.**

| **Gene Symbols** | **Forward Primers** | **Reverse Primers** |
| --- | --- | --- |
| Ap4b1 | CCCAATCTCCAGCTTACTGC | TCCTTGCCTTACTTTGCTTCA |
| Cct4 | TTATTGCAGGAGGTGGTGCT | TCGGACATTAATGCCTGTAGTTT |
| Traf4 | TGGTAGGGAGAGTGGGCTAA | GACATCCGAAAGCGGAACTA |
| Clk1 | CAGGTTCTCCATAGGGATGC | AGAAGCAGCAGCAGTCACAA |
| Setmar | GAAAGTACGCCAAGCCAGTT | CCTAACACCTCCCCAGCATA |
| Dcn | GCAGTTGGGCAAAATGACTT | TTGGGGTGTTTTTCCAGATT |
| Armc7 | GGCCAGTACCATGAGTGTCC | GCCTCTCCAGAGAGACAAGG |
| Ppp1r15a | AAAGTCCCCAAGTTTCAGGAG | ATCTCCTGTCCCCACTGATG |
| Tbx2 | CCTGGACAAGAAAGCCAAAT | TGGTCAGCTTCAGTTTGTGG |
| Gsn | CGGCTGGGATGACAACTACT | GAGTTTCCACAGGGCTTGTT |
| Rnf4 | GTGTCGCCTATGGGAGCAGT | TGGGCAAAAGCATGTAAGTG |
| Col15a1 | TTAGACACACAGGCCCTTCC | CGCTCAGCTATGACTGGGTA |
| Nfkbiz | GAGTCTCAGTTTGGGGTGGA | ACCACAGCAGCAACATCAAC |
| Rab35 | CAGTTCCCGCAGAAAGGTAG | TAGCCCTGCACTCAACAGAA |
| Mcl1 | CCCCTGGAAGAGTCACTGTC | TCACCAACGTTGTTAATTAGGG |
| Trib2 | TTCCTTTCTTCTTCCCCTGA | CATTCGCACTTTCCAATTCA |
| Tirap | GTGGCGAGGTAGGTGACATT | AGGCAGGCTCTGTTGAAGAA |
| Srfbp1 | GGTGACGACTTCTTCATTGGA | CGAGTGGAAAAACACGGATT |
| Igf1r | CTACAGCGAGGAGAACAAGC | GTGTGTCCGAGGATCAGCAG |
| Cdo1 | CCACCAGAAGCCCAAGTAGA | TGCAAAGGGTGTGTCCTAAG |
| Zfp131 | TGCCCATCAGTTTACCACAA | CGGGTAAATCAGATGCCAGT |
| Erc1 | TGAGAGAACTGAGCAGCAATG | GCCCACAAATCTCTCTCACC |
| Fiz1 | ATGGGGGAAGATCAGTAGGG | GGGGTGGGGCAATATCTTAG |
| Cldn12 | GCCTATTCAATTAGTCCCCAGA | TGAACCATTCCTTTTATCTTGC |
| Ovca2 | GGAGAGCTGCTGGACAAAGT | CATGTCACTGCCTTCACTTCA |
